# Association Between Rapid Antigen Detection Tests and Real-Time Reverse Transcription–Polymerase Chain Reaction Assay for SARS-CoV-2: A Systematic Review and Meta-Analyses

**DOI:** 10.3389/ijph.2023.1605452

**Published:** 2023-08-01

**Authors:** Yu-Pei Yang, Zhu Liduzi Jiesisibieke, Tao-Hsin Tung

**Affiliations:** ^1^ Department of Hematology, Taizhou Hospital of Zhejiang Province Affiliated to Wenzhou Medical University, Linhai, Zhejiang, China; ^2^ School of Public Health, The University of Hong Kong Li Ka Shing Faculty of Medicine, Pokfulam, Hong Kong, Hong Kong SAR, China; ^3^ Evidence-Based Medicine Center, Taizhou Hospital of Zhejiang Province Affiliated to Wenzhou Medical University, Linhai, Zhejiang, China

**Keywords:** cycle threshold, false-positive, meta-analyses, rapid antigen test, RT-PCR

## Abstract

**Objectives:** We aimed to assess the association between rapid antigen detection tests and real-time reverse transcription-polymerase chain reaction assay for severe acute respiratory syndrome coronavirus 2.

**Methods:** We searched PubMed, Cochrane Library, EMBASE, and the Web of Science from their inception to 31 May 2023. A random-effects meta-analysis was used to estimate false positives in the RADTs group, relative to those in the RT-PCR group, and subgroup analyses were conducted based on the different Ct value cut-offs (<40 or ≥40). We performed this study in accordance with the guidelines outlined in the Preferred Reporting Items for Systematic Reviews and Meta-Analyses (PRISMA).

**Results:** Fifty-one studies were included and considered to be of moderate quality. We found a satisfactory overall false positive rate (0.01, 95% CI: 0.00–0.01) for the RADTs compared to RT-PCR. In the stratified analysis, we also found that the false positive rates of the RADTs did not increase when Ct values of RT-PCR (Ct < 40, 0.01, 95% CI: 0.00–0.01; Ct ≥ 40, 0.01, 95% CI: 0.00–0.01).

**Conclusion:** In conclusion, the best available evidence supports an association between RADTs and RT-PCR. When Ct-values were analyzed using cut-off <40 or ≥40, this resulted in an estimated false positive rate of only 1%.

## Introduction

COVID-19 remains an ongoing global pandemic, and the return to pre-pandemic normalcy is still projected to be unlikely in the short run [1]. It appears that the new linages are more capable of resisting natural or vaccine-elicited immunity [2, 3]. Hence, new linages may be responsible for major re-infections and mass vaccine breakthroughs, posing overwhelming pressure on health systems [2, 3]. Frequent early testing of infectious persons, in combination with contact tracing and isolation, are key factors that mitigate transmission [4]. However, false negatives, false positives, and other inaccurate results make it even more challenging for governing authorities to set effective control strategies and make timely medical decisions [5]. Therefore, policymakers have focused on these problems and explored some cost-effective tests [6, 7].

To improve sensitivity and specificity, reverse transcription-polymerase chain reaction (RT-PCR) has been the standard method for detecting severe acute respiratory syndrome coronavirus 2 (SARS-CoV-2) since the beginning of the pandemic [8]. However, RT-PCR testing needs a remarkably long turnaround time and relies heavily on sophisticated equipment and highly trained personnel. This limits its application in mass-oriented testing campaigns [9]. Thus, the World Health Organization (WHO) recommended simple and rapid antigen detection tests (RADTs) in communities to serve the purpose of detection and contact tracing, as well as outbreak investigations [10]. This technique does not need trained experts and professional instruments and can offer results within 15 min, making it possible to identify those potentially infected with COVID-19 on time [11]. However, the WHO suggests a minimum of 80% sensitivity and 97% specificity to adopt RADTs [12], since their performance is inconsistent in diverse settings according to published research [13].

Moreover, data on the performance of self-testing with RADTs compared to RT-PCR detection of SARS-CoV-2 RNA is very limited. Previous studies have demonstrated the accuracy of RADTs [13, 14], while few have explored the relationship between RADTs and cycle threshold (Ct) value cut-offs, allowing a gap for further research. Ct values vary in different areas and are dynamically adjusted. From a clinical viewpoint, there is no consistent standardization between laboratories and assays. Thus, we aimed to explore the association between RADTs and RT-PCR for SARS-CoV-2 based on the disparity of the Ct value.

## Methods

### Literature Search

We conducted the meta-analysis following the Preferred Reporting Items for Systematic Reviews and Meta-Analyses (PRISMA) guideline. We performed a systematic search of the databases PubMed, Cochrane Library, EMBASE, and the Web of Science from their inception to 31 May 2023. The main search terms were “[(COVID-19 OR SARS-CoV-2 OR Coronavirus disease 2019 OR Novel coronavirus) AND (Rapid diagnosis* OR Rapid detection OR Rapid antigen test* OR Antigen assay) AND (false-positive OR false-positivity OR specificity OR accuracy)] AND (cycle threshold OR Ct)] (Table 1). We also searched the previous reviews for relevant studies. We registered this systematic review on PROSPERO (registration number: CRD42022351138). No language restrictions were applied.

**TABLE 1:** Search strategy until 31 May 2023 (Global, 2020–2023).

		PubMed	Embase	Cochrane	Web of science
#1	COVID-19	319,309	339,602	15,682	512,110
#2	SARS-CoV-2	118,872	117,759	6,097	105,998
#3	Coronavirus disease 2019	58,559	56,228	6,840	68,305
#4	Novel coronavirus	13,037	12,781	1,307	22,994
#5	#1 or #2 or #3 or #4	348,896	379,230	16,626	522,684
#6	Rapid diagnosis*	18,564	23,911	9,566	129,850
#7	Rapid detection	16,967	18,365	4,626	142,650
#8	Rapid antigen test*	1,161	1,422	816	12,769
#9	Antigen assay	1,018	1,520	3,760	103,490
#10	#6 or #7 or #8 or #9	36,645	44,122	16,504	413,929
#11	False-positive	55,480	75,279	2,866	96,888
#12	False-positivity	946	1,433	2,865	1,092
#13	Specificity	555,121	722,365	159,756	708,492
#14	Accuracy	543,919	687,288	27,808	1,650,362
#15	#11 or #12 or #13 or #14	1,045,545	1,328,795	179,150	3,571,103
#16	Cycle threshold	2,450	3,123	2,347	27,950
#17	ct	432,005	719,831	83,312	525,013
#18	#16 or #17	432,942	721,178	85,438	550,337
#19	#5 and #10 and #15 and #18	264	270	22	564

* means truncated word.

### Inclusion Criteria and Exclusion Criteria

We included studies evaluating the specificity or false-positivity of commercially available RADTs for diagnosis of SARS-CoV-2 infection, against RT-PCR as a reference standard. Cohort studies, nested cohort studies, and case-control or cross-sectional studies as well as randomized studies were considered. Studies carried out in various locations, targeted at individuals of any age despite presence of symptoms were pooled in our study. Studies without reporting a Ct value cut-off were excluded.

### Study Selection and Data Extraction

**FIGURE 1:**
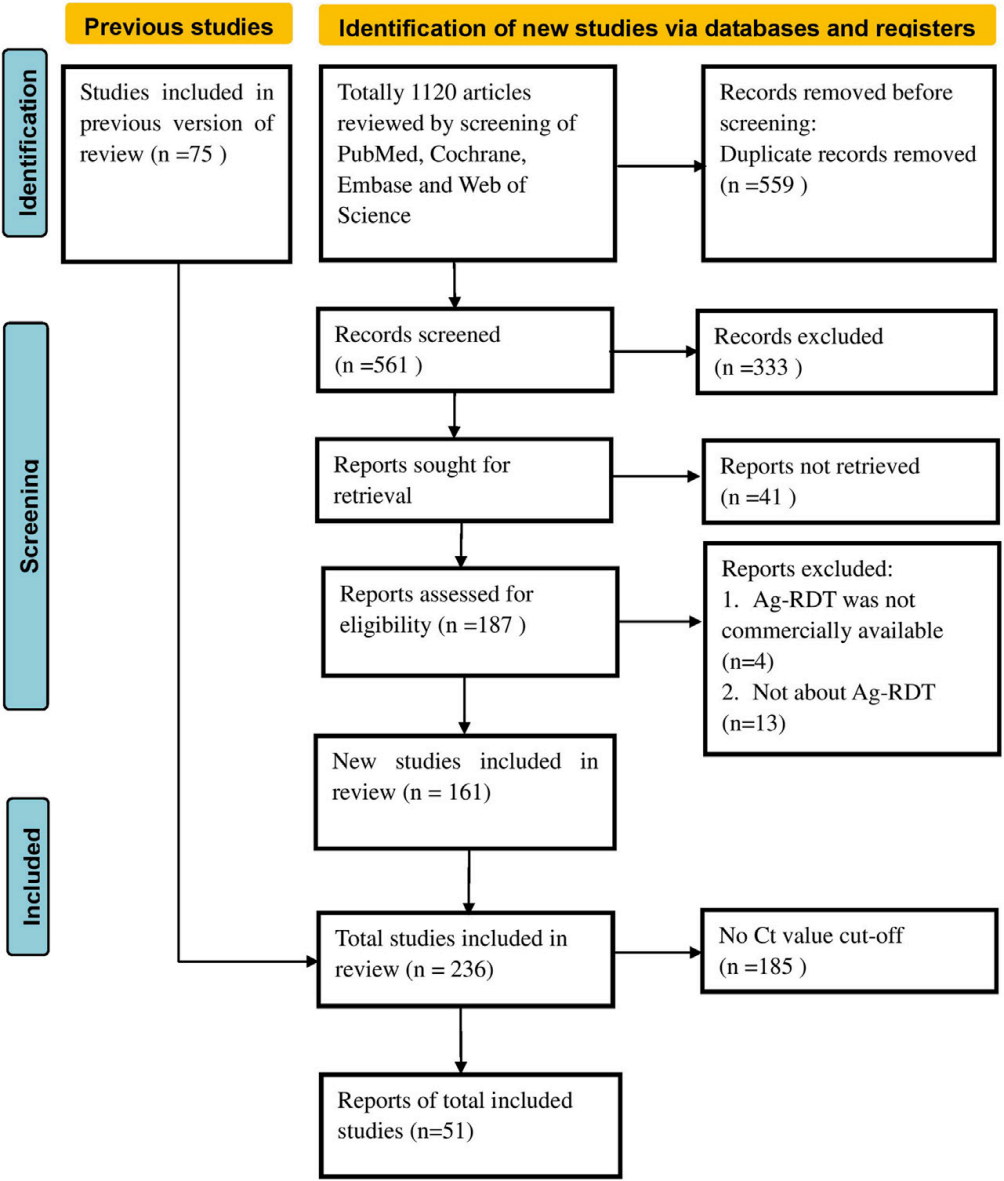
Preferred reporting items for systematic reviews and meta-analyses flow chart (Global, 2020–2023).

Two authors (Y-PY and ZJ) independently scanned the titles and abstracts of the search results in Endnote X9 to retrieve relevant records and obtained a full-text review of those eligible articles that met our inclusion criteria. Then, a third author (T-HT) was invited to settle any conflicts or disputes. Figure 1 illustrates the flowchart of the screening process. Based on the previously defined excluded criteria, 51 papers written in the English language were included in the final meta-analysis.

Afterward, data extraction was performed by an author per paper and reviewed by a second. The following data were extracted from included studies using a data-extraction form: first author, nation, sample size, sample condition, index tests, false positives, and Ct cut-off values.

### Statistical Analysis

We used STATA Version 17.0 software to conduct the meta-analysis. Subgroup analysis was conducted based on different Ct value cut-offs (<40 or ≥40). The main indicators used were percentage ratios and 95% confidence intervals (CIs) of false positives in the RADTs group relative to those in the RT-PCR group. The I^2^ statistic was used to assess the level of statistical heterogeneity, and an I^2^ value of ≥50% confirmed heterogeneity [15]. We conducted a random-effect model meta-analysis because we expected considerable clinical heterogeneity. In addition to an overall evaluation, we also conducted a sub-group meta-analysis for Ct value cut-off with <40 and those ≥40. We evaluated the quality of the evidence for each outcome using the GRADE (Grading of Recommendations Assessment, Development and Evaluation) protocols, which classified evidence as very low, low, moderate, or high [16].

## Results

The main characteristics of the included studies are summarized in Table 2.

**TABLE 2:** Characteristics of the included studies (Global, 2020–2023).

First author	Nation	Sample size	Sample condition	Index tests	Specimens	False-positive	Ct value
[17]	Japan	226	Fresh	ESPLINE SARS-CoV-2	NP	0.0%	30
[18]	Netherlands	1,367	Fresh	Abbott, Panbio	NP	0% (95% CI: 0%–0.3%)	32
		208				0% (95% CI: 0%–2.5%)	
[19]	India	677	Fresh	PathoCatch/ACCUCARE	NP	0.2% (95% CI 0.0%–0.9%)	32
[20]	India	473	Fresh	STANDARD Q, SD Biosensor	NP	0.76% (95% CI: 0.16%–2.21%)	35
[21]	Korea	175	Fresh	STANDARD Q, SD Biosensor	NP	0.0%	35
[22]	Canada	1,641	Fresh	Abbott, Panbio	NP	0.1% (95% CI 0.0%–0.5%)	35
[23]	Italy	392	Fresh	Lumipulse^®^ SARS-CoV-2 assay	NP	2.0% (95% CI 0.0%–3.0%)	35
[24]	Italy	5,136	Fresh	Panbio	NP	0.3% (95% CI 0.2%–0.6%)	35
[25]	India	1,034	Fresh	Standard™	NP	5.3% (95% CI 3.7%–7.4%)	35
[26]	Fresh	189	Fresh	Standard Q	NP	0.0%	35
[27]	India	329	Fresh	RAT kit (Zydus Cadila, India)	NP	1.11% (95% CI 0.13%–3.96%)	35
[28]	China	83	Fresh	The COVID-19 Combo Kit (Zhijiang Biotechnology Co., Ltd., Shanghai, CN)	NP	0.0%	35
[29]	France	248	Fresh	COVID‐VIRO^®^	NP	0.0%	37
[30]	Ethiopia	200	Banked	Standard Q	NP	3.0% (95% CI 0.6%–8.5%)	37
[31]	Sri Lanka	4,786	Fresh	STANDARD Q, SD Biosensor	NP	2.4% (95% CI 2.0%–3.0%)	38
		3,325		Abbott, PanBio		0.4% (95% CI 0.2%–0.8%)	
[32]	Republic of Korea	170	Banked	MARK-B	NP	1.0% (95% CI 0.1%–5.0%)	38
		170		Standard Q, SD Biosensor		0.0% (95% CI 0.0%–3.3%)	
[33]	Belgium	232	Fresh	BioSpeedia	NP	0.0%	39
				Abbott, Panbio			
[34]	Uganda	247	Fresh	BIOCREDIT	NP	1.8% (95% CI 0.4%–6.9%)	39
		194		COVID-19 Ag Respi-Strip		0.8% (95% CI 0.1%–5.5%)	
		172		PCL		10.1% (95% CI 5.1%–19.2%)	
		243		MEDsan1		0.0% (95% CI 0.0%–3.1%)	
		185		Abbott, Panbio		0.0% (95% CI 0.0%–3.6%)	
		229		Novegent		10.1% (95% CI 5.9%–16.7%)	
		263		VivaDiag™		5.9% (95% CI 3.4%–9.9%)	
[35]	Egypt	94	Banked	Artron COVID-19 Antigen test	NP	0.0%	39
[36]	Thailand	1,100	Fresh	STANDARD Q, SD Biosensor	Unclear	0.29% (95% CI: 0.06%–0.85%)	40
[37]	Korea	165	Banked	STANDARDQ, SD Biosensor	NP	4.0% (95% CI: 1.1%–9.9%)	40
[38]	China	251	Fresh	Fluorescence immunochromatographic (FIC) assay	NP	0% (95% CI 0%–8.9%)	40
[39]	Kenya	997	Fresh	NowCheck	NP/OP	2.5% (95% CI: (1.5%–3.8%)	40
[40]	Bangladesh	380	Fresh	OnSite^®^	NP	0.8% (95% CI: (0.1%–2.9%)	40
[41]	Belgium	328	Banked	COVID-19 Ag Respi-Strip	NP	0.5% (95% CI: 0.0%–2.8%)	40
[42]	Austria	392	Fresh	AMP, AMEDA Labordiagnostik GmbH, Graz, Austria	NP	0.3% (95% CI 0.0%–1.9%)	40
[43]	Chile	842	Fresh	STANDARD Q, SD Biosensor	NP	0.4% (95% CI: 0.1%–1.1%)	40
[44]	Netherlands	825	Fresh	Abbott, Panbio	NP	0% (95% CI: 0.0%–1.2%)	40
[45]	Italy	403	Fresh	Elecsys SARS- CoV- 2 antigen assay	NP	0.0% (95% CI 0.0%–1.0%)	40
[46]	Korea	296	Fresh	STANDARD Q, SD Biosensor	NP	0.0% (95% CI 0.0%–1.69%)	40
[47]	Belgium	63	Fresh	CoRDT	NP	0.0%	40
		100		HeRDT		0.0%	
[48]	Italy	169	Frozen	Lumipulse^®^ G	Saliva	2.9% (95% CI 0.6%–4.0%)	40
		127	Fresh			0.0% (95% CI 0.0%–7.9%)	
[49]	Belgium	199	Fresh and banked	GSD NovaGen	NP	14.3% (95% CI 4.5%–24.1%)	40
		199		Cassette, BioRad		0.0%	
		199		Aegle, LumiraDx		0.0%	
[50]	Madagascar	200	Fresh	Standard Q, SD Biosensor	NP	0.0%	40
[51]	Chile	64	Banked	Sofia	NP/OP	3.1% (95% CI 0.6%–15.7%)	40
		64		SD Biosensor, Standard F		3.1% (95% CI 0.6%–15.7%)	
[52]	Republic of Korea	141	Banked	AFIAS	NP	0.2%	40
		156		AFIAS		0.0% (95% CI 0.0%–2.0%)	
		167		AFIAS			
		200		ichroma™			
[53]	Brazil	127	Fresh	Abbott, Panbio	NP	1.8% (95% CI 1.2%–4.0%)	40
[54]	United States	1,384	Fresh	Becton, BD Veritor	NP	1.2% (95% CI 0.7%–1.9%)	40
[55]	Chile	109	Banked	RapiGEN, Biocredit	NP/OP	0.0% (95% CI 0.0%–11.6%)	40
		19		Liming Bio		10.0% (95% CI 1.8%–40.4%)	
		109		Savant Biotechnology Co., Beijing		0.0% (95% CI 0.0%–11.0%)	
		111		Shenzhen Bioeasy Biotechnology		0.0% (95% CI 0.0%–11.2%)	
[56]	Belgium	148	Fresh	Coris BioConcept, COVID-19 Ag Respi-Strip	NP	0.0% (95% CI 0.0%–8.4%)	40
[57]	Belgium	414	Banked	DiaSorin, LIAISON	NP	0.0% (95% CI 0.0%–1.7%)	40
[58]	Italy	226	Banked	Fujirebio, Lumipulse G	NP	7.9% (95% CI 6.6%–9.3%)	40
		1,738	Fresh			8.4% (95% CI 4.3%–14.5%)	
[59]	Thailand	454	Banked	SDBiosensor/Roche, Standard Q	NP/OP	1.3% (95% CI 0.4%–2.9%)	40
[60]	Indonesia	313	Fresh	Ag-RDT kits	NP/OP	0.0%	40
[61]	Austria	175	Fresh	AMP rapid test SARS-CoV-2 Ag	NP	0.0% (95% CI 0.0%–3.3%)	40
[62]	Bangladesh	260	Fresh	BD Veritor, Standard Q	NP	0.0% (95% CI 0.0%–3.0%)	40
[63]	Netherlands	683	Fresh	Panbio™	NP	0.0% (95% CI 0.0%–0.6%)	40
[64]	Kenya	2,245	Fresh	Panbio™	NP	1.5% (95% CI 1.0%–2.2%)	40
[65]	Japan	100	Banked	STANDARD Q	NP	0.0% (95% CI 0.0%–17.0%)	40
[66]	Serbia	120	Fresh	Standard Q	NP	0.0% (95% CI 0.0%–4.7%)	41
[67]	Korea	370	Fresh	Xpert Xpress	NP	4.6%	45

NP, nasopharyngeal; OP, oropharyngeal; AN, anterior nasal.

**FIGURE 2:**
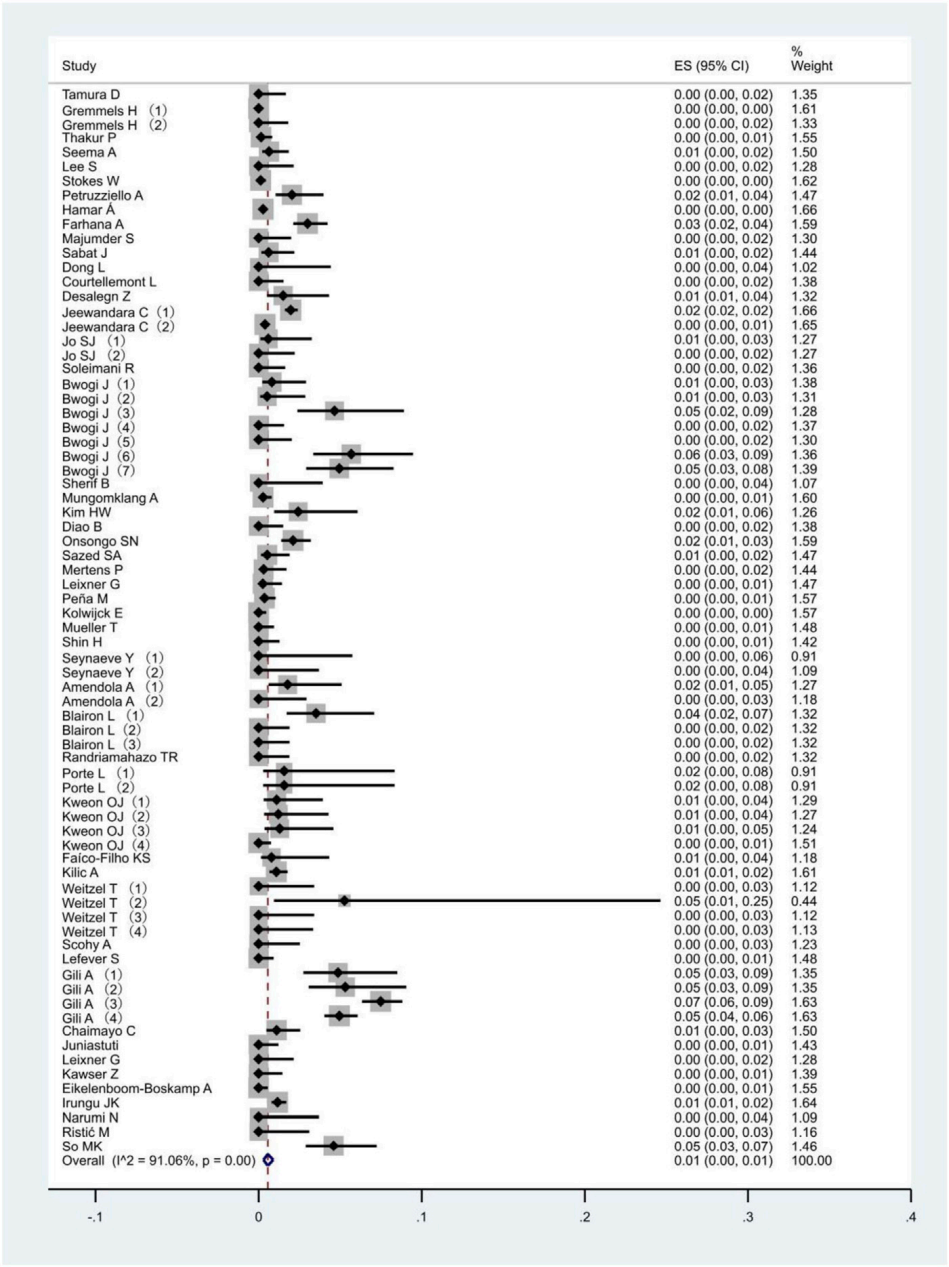
Meta-analysis of the false positive in all people (Global, 2020–2023).

**FIGURE 3:**
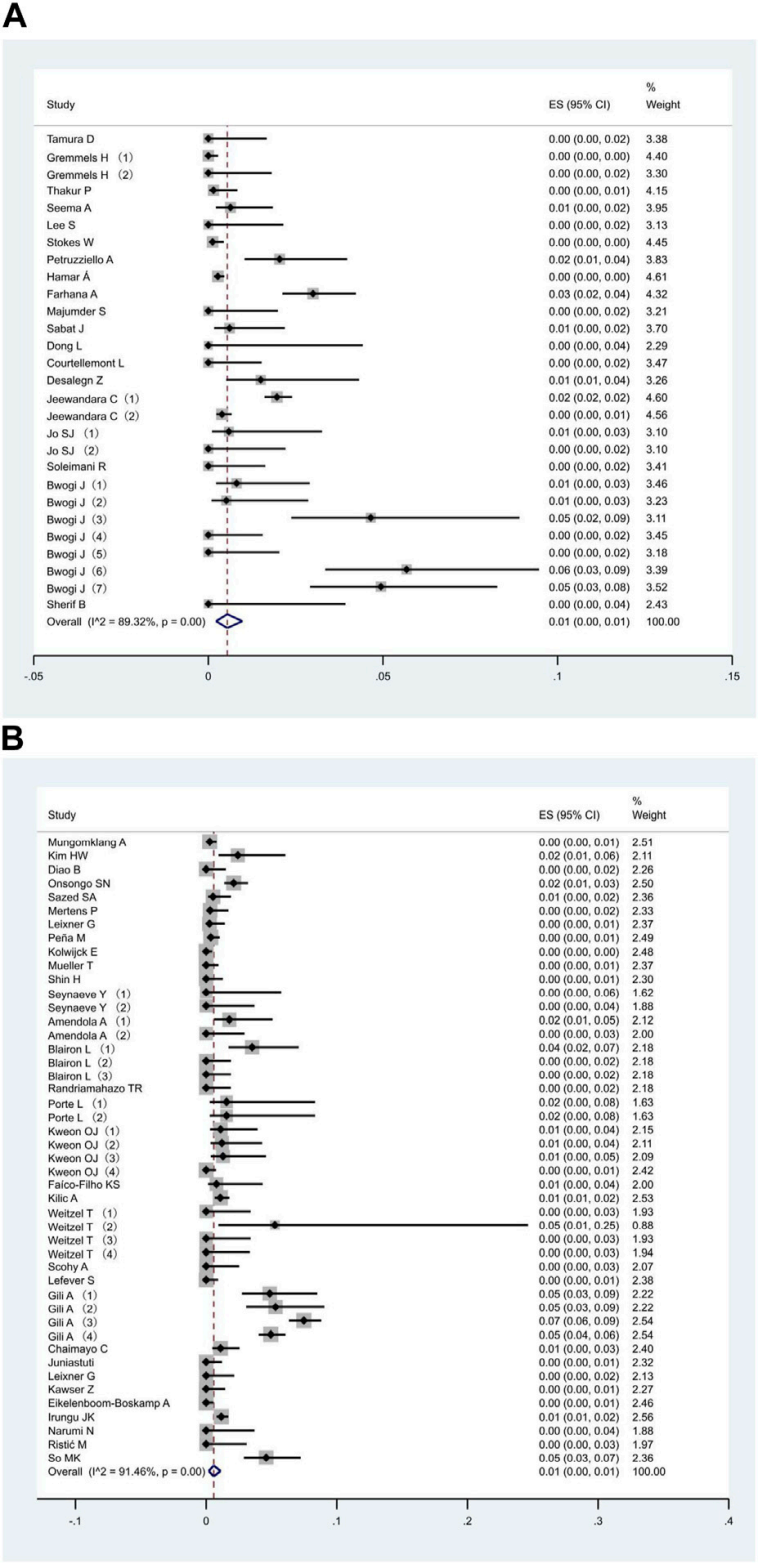
Meta-analysis of the false positive in people with Ct value cut-off <40 **(A)** and ≥40 **(B)** (Global, 2020–2023).

All 51 included studies [17–67] evaluated the false positive rates of RADTs with respect to the standard RT- PCR while reporting a Ct cut-off value. The most common sample type evaluated was NP (44/51), followed by mixed NP/OP (5/51). We divided the 51 studies into 74 data sets. The overall pooled estimates of false positive rates of RADTs were 1% (95% CI: 0.00–0.01) compared to RT-PCR (Figure 2). In the stratified analysis, a similar pattern was observed when Ct-values were analyzed using cut-off <40 or ≥40, resulting in an estimated false positive rate of 1% (95% CI: 0.00–0.01) and 1% (95% CI: 0.00–0.01), respectively (Figures 3A,B).

The summary of findings and the GRADE assessment for each outcome is presented in Table 3. The quality of evidence from the included studies was initially judged to be moderate due to imprecision.

**TABLE 3:** GRADE summary of findings (Global, 2020–2023).

False positive rates					
Patient or population					
Setting: Netherlands, India, Korea, Canada, France, Sri Lanka, Republic of Korea, Belgium, Uganda, Thailand, Bangladesh, Austria, Chile, Italy, Madagascar, United States, Serbia					
Intervention*: RADTs					
**Outcomes**	**Event**	**Total**	**Effect size (95% CI)**	**Quality of the evidence (GRADE)**	**Comments**
False positive rates	209	22,688	0.01 (0.00, 0.01)	⊕⊕⊕○ Moderate	CT < 40
False positive rates	357	19,436	0.01 (0.00, 0.01)	⊕⊕⊕○ Moderate	CT ≥ 40
False positive rates	566	42,124	0.01 (0.00, 0.01)	⊕⊕⊕○ Moderate	Overall

*The risk in the intervention group (and its 95% confidence interval) is based on the assumed risk in the comparison group and the relative effect of the intervention (and its 95% CI).

CI: Confidence interval.

GRADE working group grades of evidence

High quality: We are very confident that the true effect lies close to that of the estimate of the effect.

Moderate quality: We are moderately confident in the effect estimate: The true effect is likely to be close to the estimate of the effect, but there is a possibility that it is substantially different.

Low quality: Our confidence in the effect estimate is limited: The true effect may be substantially different from the estimate of the effect.

Very low quality: We have very little confidence in the effect estimate: The true effect is likely to be substantially different from the estimate of effect.

## Discussion

### Clinical Implications

Although COVID-19 is not a chronic disease, it meets the Wilson criteria for screening due to the following facts: it is an important health problem; its natural history and side effects are well understood; there is a recognizable latent or early symptomatic stage; a test is easy to perform and its acceptably, accurately, reliably, sensitively and specifically easy to interpret; an accepted treatment is recognized for the disease; treatment is more effective if started early; a policy on who should be treated has been in execution; timely diagnosis and treatment are cost-effective, and case finding should be a continuous process [68].

To our knowledge, this is the first systematic review and meta-analysis that explores the relationship between RADTs and Ct value cut-offs. We integrated data from 51 studies to evaluate the false positive rates of RADTs. We found an overall satisfactory false positive rate (0.01, 95% CI: 0.00–0.01) for RADTs compared to RT-PCR. In the stratified analysis, we also found that false positive rates of RADTs did not increase when Ct values of RT-PCR increased (Ct < 40, 0.01, 95% CI: 0.00–0.01; Ct ≥ 40, 95% CI: 0.00–0.01).

Amplification of genomic sequence of RT-PCR is measured in Ct values. Reporting this Ct value or calculating viral load aids in interpretation and clinical decision-making [69]. However, there are controversies on how thresholds for infectivity shall be defined [70]. A previously published meta-analysis [71] showed that

RADTs present high sensitivity and specificity in detecting COVID-19 but not in exploring the relationship between Ct values and sensitivity/specificity. Since it is difficult to standardize Ct values across different systems and research teams, we could not compare the results between the studies included with different Ct values [71]. Many qPCR assays introduced a Ct cut-off of 40 for infectivity, allowing the detection of very few starting RNA molecules [69]. Our study was able to prove further the effectiveness of the association between RADTs and RT-PCR in different groups of Ct-Values.

Furthermore, it is of great necessity to carry out mass testing in communities as a major strategy for transmission control to minimize the spread of the infection because statistics show that those who never develop symptoms or are still in the before-symptom-onset stage can be quite contagious [72]. Therefore, self-testing using RADTs is vital in low-cost, resource-saving infection control [73]. Meanwhile, swab sampling from the oral or anterior nasal (AN) is less invasive and more efficient in improving compliance and testing frequency. Therefore, if such tests are adopted in community screening, especially in densely populated sites like schools and universities, rest homes, clinics, and prisons, they may help to reduce or even eliminate transmission significantly [58]. As it is easy to perform, all individuals can collect samples independently. This would eventually reduce the quarantine time for the potentially infected [69] and allow individuals to return to their regular work as early as possible. But the prerequisite for a reliable and validated SARS-CoV-2 test is compliance with negative and traditional physical preventive measures like face masks and social distancing that must be strictly observed [74]. Besides, such diagnostic capability would also enable policymakers to evaluate and adjust restrictive rules like quarantine duration and business shutdown period to ensure quick recovery of the economy.

The clinicians could also adjust discharge criteria according to Ct values and determine when the patient can discontinue isolation (which will shorten the hospitalization and isolation time of patients further and avoid unnecessary isolation treatment and waste of medical resources). The adjustment of Ct-values has implications in public health screening, enabling contact tracers to focus on persons who are most likely to be infectious. Reduced screening performance induced by the low sensitivity of RADTs makes it essential to guarantee effective repetition in close screening [75, 76].

### Clinical Practice

The COVID-19 pandemic threatened the effective management of hospital risk. Health care providers should respond timely and effectively to rapidly changing regulations and guidelines [77]. During this period, hospitals need to care for COVID-19 patients, but also those with other diseases. The lower false positive rate and costs of RADTs imply that early and suitable detection was effective.

Nevertheless, the consequences of failing to identify and separate those COVID-19 cases affect the quality of care [78, 79]. Cases that were negative on the RADTs but had clinical symptoms were sent for further RT-PCR testing for timely identification of COVID-19 cases.

RT-PCR is the most important indicator for the clinical diagnosis of COVID-19. However, since it requires substantial manpower and time, rapid and accurate laboratory diagnosis technology is very important. The results of this study implied that RADTs could be considered an alternative for the rapid triage of patients. Although manufacturers’ instructions vary, our study provides guidance in the real world: RADTs are easy to conduct, do not require expert knowledge, and the results are obtained within a few minutes, saving time and money. If suspicious patients had similar clinical symptoms but were negative for RCTs, they were further tested with RT-PCR. In addition, RADTs are also suitable for epidemiological analysis, such as group epidemic monitoring and contact tracing. Such integrated strategies could significantly enhance prevention.

RADTs is not only a reasonably inexpensive, simple test with quick results and more accessibility to patients, but is also an important tool that might be more useful in cutting off the chain of transmission by rapidly identifying positive and previous cases, discovering vast numbers of asymptomatic carriers who often migrate from one location to another [80, 81]. In addition, RDTs can provide additional seroepidemiological data and aetiological diagnosis to determine the magnitude of COVID-19 spread within a population [80]. Thus, previous studies indicated that RADTs were recommended for the early detection of patients suspected of having COVID-19 at the peripheral level of the health system and outside hospital settings in low- and middle-income countries [80–82], where there is little access to molecular tests.

### Heterogeneity of Meta-Analysis

In the meta-analysis, heterogeneity exists if the sample estimates for the population risk were of different magnitudes [83]. In this study, we used the random effect model when I^2^ statistics were 89.32%, 91.46%, and 91.02% for Ct values < 40, Ct values ≥ 40, and overall, respectively. Because of the existence of significant heterogeneity in false positive rates, it is important to assess heterogeneity in the meta-analysis. This could be caused by various factors, including population characteristics, study design, sample quality, antigen test manufacturers’ instructions, and Ct cut-off values [7]. We aggregated studies that explicitly reported a Ct cut-off value, but heterogeneity in the results was inevitable.

### Methodological Considerations

The strengths of this study are as follows: First, we included all relevant studies from a global database, which are accepted with a relatively high level of evidence. Second, we excluded studies with no control groups to increase comparability and decrease possible heterogeneity. This is because a larger publication bias may exist without control groups. Third, a subgroup meta-analysis was performed to analyze the real association that controlled the independent Ct values. We analyzed the separate effects of the association between RADTs and RT-PCR based on different CT values.

However, there are still several limitations that should be noted when interpreting the findings of this meta-analysis. Firstly, because sources of reagents are very complex and no single diagnosis standard exists, the bias estimated is inevitable. Secondly, ordinary meta-analyses on efficacy render high-quality evidence from randomized controlled trials only. However, it is impossible to randomize people into “RADTs” and “RT-PCR” categories. Thirdly, few studies considered other potential confounding factors. Fourthly, the variable studied is only at Ct values in a rough range with a cut-off of <40 or ≥40, and it has failed to classify CT value cut-offs in more groups, such as below 29, 30–37, 38–40, and above 40, and add any other factors, for example the type of RADTs and RT-PCRs, the day the patient was examined, the type of reagent, the time to complete the examination, etc. Fifthly, when using the GRADE approach to evaluate the quality of the evidence for each outcome, the current evidence from all selected studies was moderate in imprecision. In the future, more comprehensive studies are recommended to improve the quality of evidence. Finally, only English papers were included, it was very difficult to explore the disparity of languages for the relationship between RADTs and RT-PCRs.

### Conclusion

In conclusion, the best available evidence supports an association between RADTs and RT-PCR. When Ct-values were analyzed using cut-off <40 or ≥40, this resulted in an estimated false positive rate of only 1%. However, it is limited, and more trials are warranted. Further studies regarding subgroups according to sex and age are essential to clarifying the subgroup effect.

## References

[1] Khandia R Singhal S Alqahtani T. Emergence of SARS-CoV-2 Omicron (B.1.1.529) Variant, Salient Features, High Global Health Concerns and Strategies to Counter it Amid Ongoing COVID-19 Pandemic. Environ Res (2022) 209:112816. 10.1016/j.envres.2022.112816 35093310 PMC8798788

[2] Planas D Saunders N Maes P. Considerable Escape of SARS-CoV-2 Omicron to Antibody Neutralization. Nature (2022) 602(7898):671–5. 10.1038/s41586-021-04389-z 35016199

[3] Taylor L. Covid-19: Omicron Drives Weekly Record High in Global Infections. BMJ (2022) 376:o66. 10.1136/bmj.o66 35017144

[4] Contreras S Dehning J Loidolt M Zierenberg J Paul Spitzner F Jorge H The Challenges of Containing SARS-CoV-2 via Test-Trace-And-Isolate. Nat Commun (2021) 12:378. 10.1038/s41467-020-20699-8 33452267 PMC7810722

[5] Li Z Yi Y Luo X Xiong N Liu Y Li S Development and Clinical Application of a Rapid IgM-IgG Combined Antibody Test for SARS-CoV-2 Infection Diagnosis. J Med Virol (2020) 92(9):1518–24. 10.1002/jmv.25727 32104917 PMC7228300

[6] Ilkhani H Hedayat N Farhad S. Novel Approaches for Rapid Detection of COVID-19 During the Pandemic: A Review. Anal Biochem (2021) 634:114362. 10.1016/j.ab.2021.114362 34478703 PMC8406551

[7] Dinnes J Deeks JJ Berhane S. Rapid, Point-Of-Care Antigen and Molecular-Based Tests for Diagnosis of SARS-CoV-2 Infection. Cochrane Database Syst Rev (2021) 3(3):CD013705. 10.1002/14651858.CD013705.pub2 33760236 PMC8078597

[8] García-Fiñana M Hughes DM Cheyne CP. Performance of the Innova SARS-CoV-2 Antigen Rapid Lateral Flow Test in the Liverpool Asymptomatic Testing Pilot: Population Based Cohort Study. BMJ (2021) 374:n1637. 10.1136/bmj.n1637 34230058 PMC8259455

[9] Corman VM Landt O Kaiser M Molenkamp R Meijer A Chu DK Detection of 2019 Novel Coronavirus (2019-nCoV) by Real-Time RT-PCR. Euro Surveill (2020) 25(3):2000045. 10.2807/1560-7917.ES.2020.25.3.2000045 31992387 PMC6988269

[10] World Health Organization. Antigen-Detection in the Diagnosis of SARS-CoV- 2 Infection: Interim Guidance (2021). Available from: https://apps.who.int/iris/handle/10665/345948 (Accessed December 8, 2021).

[11] Aranaz-Andrés JM Chávez ACF Laso AM. Analysis of the Diagnostic Accuracy of Rapid Antigenic Tests for Detection of SARS-CoV-2 in Hospital Outbreak Situation. Eur J Clin Microbiol Infect Dis (2022) 41(2):305–12. 10.1007/s10096-021-04346-8 34792699 PMC8600491

[12] Peeling RW Olliaro PL Boeras DI. Scaling up COVID-19 Rapid Antigen Tests: Promises and Challenges. Lancet Infect Dis (2021) 21:e290–e295. 10.1016/S1473-3099(21)00048-7 33636148 PMC7906660

[13] Brümmer LE Katzenschlager S McGrath S Schmitz S Gaeddert M Erdmann C Accuracy of Rapid Point-Of-Care Antigen-Based Diagnostics for SARS-CoV-2: An Updated Systematic Review and Meta-Analysis with Meta-Regression Analyzing Influencing Factors. Plos Med (2022) 19(5):e1004011. 10.1371/journal.pmed.1004011 35617375 PMC9187092

[14] Yang YP Huang LL Pan SJ Xu D Jiesisibieke ZL Tung TH. False-Positivity Results in Rapid Antigen Tests for SARS-CoV-2: An Umbrella Review of Meta-Analyses and Systematic Reviews. Expert Rev Anti Infect Ther (2022) 20(7):1005–13. 10.1080/14787210.2022.2070152 35452591

[15] Higgins JP Thompson SG Deeks JJ. Measuring Inconsistency in Meta-Analyses. BMJ (2003) 327(7414):557–60. 10.1136/bmj.327.7414.557 12958120 PMC192859

[16] Guyatt GH Oxman AD Vist GE Kunz R Falck-Ytter Y Alonso-Coello P GRADE: An Emerging Consensus on Rating Quality of Evidence and Strength of Recommendations. BMJ (2008) 336(7650):924–6. 10.1136/bmj.39489.470347.AD 18436948 PMC2335261

[17] Tamura D Yamagishi H Morisawa Y Mato T Nunomiya S Maehara Y Diagnostic Accuracy of a Novel SARS CoV-2 Rapid Antigen Test and Usefulness of Specimens Collected From the Anterior Nasal Cavity. Int J Infect Dis (2022) 124:199–205. 10.1016/j.ijid.2022.09.018 36122672 PMC9481473

[18] Gremmels H Winkel BMF Schuurman R Rosingh A Rigter NAM Rodriguez O Real-Life Validation of the Panbio™ COVID-19 Antigen Rapid Test (Abbott) in Community-Dwelling Subjects with Symptoms of Potential SARS-CoV-2 Infection. EClinicalMedicine (2021) 31:100677. 10.1016/j.eclinm.2020.100677 33521610 PMC7832943

[19] Thakur P Saxena S Manchanda V Rana N Goel R Arora R. Utility of Antigen-Based Rapid Diagnostic Test for Detection of SARS-CoV-2 Virus in Routine Hospital Settings. Lab Med (2021) 52(6):e154–e158. 10.1093/labmed/lmab033 33928384 PMC8135470

[20] Seema A Naziya Z Asif J Salim Khan SM. Diagnostic Accuracy of STANDARD Q COVID-19 Antigen Detection Kit in Comparison with RT-PCR Assay Using Nasopharyngeal Samples in India. J Clin Diagn Res (2022) 16(1):DC01–DC05. 10.7860/JCDR/2022/52286.15825

[21] Lee S Widyasari K Yang HR Jang J Kang T Kim S. Evaluation of the Diagnostic Accuracy of Nasal Cavity and Nasopharyngeal Swab Specimens for SARS-CoV-2 Detection via Rapid Antigen Test According to Specimen Collection Timing and Viral Load. Diagnostics (Basel) (2022) 12(3):710. 10.3390/diagnostics12030710 35328263 PMC8947492

[22] Stokes W Berenger BM Portnoy D Scott B Szelewicki J Singh T Clinical Performance of the Abbott Panbio with Nasopharyngeal, Throat, and Saliva Swabs Among Symptomatic Individuals with COVID-19. Eur J Clin Microbiol Infect Dis (2021) 40(8):1721–6. 10.1007/s10096-021-04202-9 33742322 PMC7979467

[23] Petruzziello A Sabatino R Catapane LA De Falco C Petti A Tripaldelli E Analytical Performance Evaluation of Lumipulse® SARS-CoV-2 Antigen Assay in 392 Asymptomatic Patients. J Clin Lab Anal (2023) 37(6):e24867. 10.1002/jcla.24867 36972465 PMC10156099

[24] Hamar Á Filipánits K Váradi A Váradi-Rácz R Gellén HO Futács K Diagnostic Accuracy of SARS-CoV-2 Panbio™ Rapid Antigen Diagnostic Tests in a 4,440-Case Clinical Follow-Up. Front Med (Lausanne) (2022) 9:908127. 10.3389/fmed.2022.908127 35983094 PMC9380887

[25] Farhana A Zahoor D Wani S Khan RA Nasir R Kanth F. Diagnostic Utility and Performance of Rapid Antigen Test in SARS CoV- 2 in Symptomatic and Asymptomatic Patients During the Second Pandemic Wave in Kashmir, North India. Indian J Med Microbiol (2022) 40(4):572–6. 10.1016/j.ijmmb.2022.06.007 35787334 PMC9249411

[26] Majumder S Chakrabarti A Das B Sarkar A Majumdar T. Comparison of SARS-CoV-2 Diagnosis by Rapid Antigen Detection Kit with RT-qPCR in a Tertiary Care Setup in North Eastern India. Indian J Med Microbiol (2023) 42:12–6. 10.1016/j.ijmmb.2022.12.016 36967208 PMC9851292

[27] Sabat J Subhadra S Rath S Ho LM Satpathy T Pattnaik D A Comparison of SARS-CoV-2 Rapid Antigen Testing with Realtime RT-PCR Among Symptomatic and Asymptomatic Individuals. BMC Infect Dis (2023) 23(1):87. 10.1186/s12879-022-07969-0 36759762 PMC9909630

[28] Dong L Li WF Jiang Y. Performance Evaluation of Antigen Detection Rapid Diagnostic Test (Ag-RDT) for COVID-19 Diagnosis in a Primary Healthcare Center During the Shanghai COVID-19 Quarantine Period. Virol J (2022) 19(1):140. 10.1186/s12985-022-01871-6 36050725 PMC9434095

[29] Courtellemont L Guinard J Guillaume C Giaché S Rzepecki V Seve A High Performance of a Novel Antigen Detection Test on Nasopharyngeal Specimens for Diagnosing SARS-CoV-2 Infection. J Med Virol (2021) 93(5):3152–7. 10.1002/jmv.26896 33615487 PMC8014580

[30] Desalegn Z Sebre S Yohannes M Seman A Shiferaw W Ademe M Comparison of the Diagnostic Performance of a Rapid Antigen Test with Real-Time Polymerase Chain Reaction for Detection of SARS-CoV-2 Among Patients Diagnosed with COVID-19 at Selected Hospitals in Addis Ababa, Ethiopia. Infect Drug Resist (2022) 15:4299–305. 10.2147/IDR.S353844 35965848 PMC9365320

[31] Jeewandara C Guruge D Pushpakumara PD Madhusanka D Jayadas TT Chaturanga IP Sensitivity and Specificity of Two WHO Approved SARS-CoV2 Antigen Assays in Detecting Patients with SARS-CoV2 Infection. BMC Infect Dis (2022) 22(1):276. 10.1186/s12879-022-07240-6 35317731 PMC8938642

[32] Jo SJ Shin SH Kim J Lee S Lee J. Evaluation of the Clinical Performance of a Magnetic Force-Assisted Electrochemical Immunoassay for the Detection of SARS-CoV-2 Antigens. PLoS One (2021) 16(10):e0258394. 10.1371/journal.pone.0258394 34618868 PMC8496795

[33] Soleimani R Deckers C Huang TD Bogaerts P Evrard S Wallemme I Rapid COVID-19 Antigenic Tests: Usefulness of a Modified Method for Diagnosis. J Med Virol (2021) 93(9):5655–9. 10.1002/jmv.27094 34009649 PMC8242554

[34] Bwogi J Lutalo T Tushabe P Bukenya H Eliku JP Ssewanyana I Field Evaluation of the Performance of Seven Antigen Rapid Diagnostic Tests for the Diagnosis of SARs-CoV-2 Virus Infection in Uganda. PLoS One (2022) 17(5):e0265334. 10.1371/journal.pone.0265334 35536792 PMC9089886

[35] Sherif B Hafez HM Abdelhalim MR Elwafa MAZA Wahba NS Hamdy P. Evaluation of Diagnostic Performance of SARS-CoV-2 Detection Kits: a Comparative Study. Beni Suef Univ J Basic Appl Sci (2023) 12(1):17. 10.1186/s43088-023-00360-1 36819293 PMC9924908

[36] Mungomklang A Trichaisri N Jirachewee J Sukprasert J Tulalamba W Viprakasit V. Limited Sensitivity of a Rapid SARS-CoV-2 Antigen Detection Assay for Surveillance of Asymptomatic Individuals in Thailand. Am J Trop Med Hyg (2021) 105(6):1505–9. 10.4269/ajtmh.21-0809 34634778 PMC8641330

[37] Kim HW Park M Lee JH. Clinical Evaluation of the Rapid STANDARD Q COVID-19 Ag Test for the Screening of Severe Acute Respiratory Syndrome Coronavirus 2. Ann Lab Med (2022) 42(1):100–4. 10.3343/alm.2022.42.1.100 34374355 PMC8368224

[38] Diao B Wen K Zhang J Chen J Han C Chen Y Accuracy of a Nucleocapsid Protein Antigen Rapid Test in the Diagnosis of SARS-CoV-2 Infection. Clin Microbiol Infect (2021) 27(2):289.e1–289.e4. 10.1016/j.cmi.2020.09.057 PMC7534827 33031947

[39] Onsongo SN Otieno K van Duijn S Adams E Omollo M Odero IA Performance of a Rapid Antigen Test for SARS-CoV-2 in Kenya. Diagn Microbiol Infect Dis (2022) 102(2):115591. 10.1016/j.diagmicrobio.2021.115591 34920265 PMC8558097

[40] Sazed SA Kibria MG Hossain MS Zamil MF Adhikary PC Ahmed D Clinical Evaluation of a New Antigen-Based COVID-19 Rapid Diagnostic Test From Symptomatic Patients. Diagnostics (Basel) (2021) 11(12):2300. 10.3390/diagnostics11122300 34943537 PMC8699944

[41] Mertens P De Vos N Martiny D Jassoy C Mirazimi A Cuypers L Development and Potential Usefulness of the COVID-19 Ag Respi-Strip Diagnostic Assay in a Pandemic Context. Front Med (Lausanne) (2020) 7:225. 10.3389/fmed.2020.00225 32574326 PMC7227790

[42] Leixner G Voill-Glaninger A Bonner E Kreil A Zadnikar R Viveiros A. Evaluation of the AMP SARS-CoV-2 Rapid Antigen Test in a Hospital Setting. Int J Infect Dis (2021) 108:353–6. 10.1016/j.ijid.2021.05.063 34087486 PMC8168346

[43] Peña M Ampuero M Garcés C Gaggero A García P Velasquez MS Performance of SARS-CoV-2 Rapid Antigen Test Compared with Real-Time RT-PCR in Asymptomatic Individuals. Int J Infect Dis (2021) 107:201–4. 10.1016/j.ijid.2021.04.087 33945868 PMC8088036

[44] Kolwijck E Brouwers-Boers M Broertjes J van Heeswijk K Runderkamp N Meijer A Validation and Implementation of the Panbio COVID-19 Ag Rapid Test for the Diagnosis of SARS-CoV-2 Infection in Symptomatic Hospital Healthcare Workers. Infect Prev Pract (2021) 3(2):100142. 10.1016/j.infpip.2021.100142 34316580 PMC8050397

[45] Mueller T Kompatscher J La Guardia M. Diagnostic Performance of the Elecsys SARS-CoV-2 Antigen Assay in the Clinical Routine of a Tertiary Care Hospital: Preliminary Results From a Single-Center Evaluation. J Clin Lab Anal (2021) 35(8):e23906. 10.1002/jcla.23906 34251047 PMC8373346

[46] Shin H Lee S Widyasari K Yi J Bae E Kim S. Performance Evaluation of STANDARD Q COVID-19 Ag Home Test for the Diagnosis of COVID-19 During Early Symptom Onset. J Clin Lab Anal (2022) 36(6):e24410. 10.1002/jcla.24410 35441745 PMC9110955

[47] Seynaeve Y Heylen J Fontaine C Maclot F Meex C Diep AN Evaluation of Two Rapid Antigenic Tests for the Detection of SARS-CoV-2 in Nasopharyngeal Swabs. J Clin Med (2021) 10(13):2774. 10.3390/jcm10132774 34202731 PMC8267731

[48] Amendola A Sberna G Lalle E Colavita F Castilletti C Menchinelli G Saliva is a Valid Alternative to Nasopharyngeal Swab in Chemiluminescence-Based Assay for Detection of SARS-CoV-2 Antigen. J Clin Med (2021) 10(7):1471. 10.3390/jcm10071471 33918294 PMC8038133

[49] Blairon L Cupaiolo R Thomas I Piteüs S Wilmet A Beukinga I Efficacy Comparison of Three Rapid Antigen Tests for SARS-CoV-2 and How Viral Load Impact Their Performance. J Med Virol (2021) 93(10):5783–8. 10.1002/jmv.27108 34050945 PMC8242364

[50] Randriamahazo TR Andrianarivelo AM Rakotoarivo AT Raheritiana TM Rakotovao LA Randriamanantany ZA Evaluation of Antigen-Based Rapid Detection Test for the Diagnosis of SARS CoV-2 in Low-Income Countries. J Virol Methods (2022) 300:114409. 10.1016/j.jviromet.2021.114409 34896454 PMC8654736

[51] Porte L Legarraga P Iruretagoyena M Vollrath V Pizarro G Munita J Evaluation of Two Fluorescence Immunoassays for the Rapid Detection of SARS-CoV-2 Antigen-New Tool to Detect Infective COVID-19 Patients. PeerJ (2021) 9:e10801. 10.7717/peerj.10801 33552746 PMC7827970

[52] Kweon OJ Lim YK Kim HR Choi Y Choi SH Chung JW Evaluation of Rapid SARS-CoV-2 Antigen Tests, AFIAS COVID-19 Ag and Ichroma COVID-19 Ag, with Serial Nasopharyngeal Specimens From COVID-19 Patients. PLoS One (2021) 16(4):e0249972. 10.1371/journal.pone.0249972 33831118 PMC8031412

[53] Faíco-Filho KS Júnior FEF Moreira LVL Lins PRG Justo AFO Bellei N. Evaluation of the Panbio™ COVID-19 Ag Rapid Test at an Emergency Room in a Hospital in São Paulo, Brazil. Braz J Infect Dis (2022) 26(2):102349. 10.1016/j.bjid.2022.102349 35358471 PMC8934711

[54] Kilic A Hiestand B Palavecino E. Evaluation of Performance of the BD Veritor SARS-CoV-2 Chromatographic Immunoassay Test in Patients with Symptoms of COVID-19. J Clin Microbiol (2021) 59(5):e00260-21. 10.1128/JCM.00260-21 33637583 PMC8091854

[55] Weitzel T Legarraga P Iruretagoyena M Pizarro G Vollrath V Araos R Comparative Evaluation of Four Rapid SARS-CoV-2 Antigen Detection Tests Using Universal Transport Medium. Trav Med Infect Dis (2021) 39:101942. 10.1016/j.tmaid.2020.101942 PMC7708826 33278609

[56] Scohy A Anantharajah A Bodéus M. Low Performance of Rapid Antigen Detection Test as Frontline Testing for COVID-19 Diagnosis. J Clin Virol (2020) 129:104455. 10.1016/j.jcv.2020.104455 32485618 PMC7240272

[57] Lefever S Indevuyst C Cuypers L Dewaele K Yin N Cotton F Comparison of the Quantitative DiaSorin Liaison Antigen Test to Reverse Transcription-PCR for the Diagnosis of COVID-19 in Symptomatic and Asymptomatic Outpatients. J Clin Microbiol (2021) 59(7):e0037421. 10.1128/JCM.00374-21 33849953 PMC8218764

[58] Gili A Paggi R Russo C Cenci E Pietrella D Graziani A Evaluation of Lumipulse® G SARS-CoV-2 Antigen Assay Automated Test for Detecting SARS-CoV-2 Nucleocapsid Protein (NP) in Nasopharyngeal Swabs for Community and Population Screening. Int J Infect Dis (2021) 105:391–6. 10.1016/j.ijid.2021.02.098 33647511 PMC7908845

[59] Chaimayo C Kaewnaphan B Tanlieng N. Rapid SARS-CoV-2 Antigen Detection Assay in Comparison with Real-Time RT-PCR Assay for Laboratory Diagnosis of COVID-19 in Thailand. Virol J (2020) 17(1):177. 10.1186/s12985-020-01452-5 33187528 PMC7665091

[60] Juniastuti Furqoni AH Amin M Restifan YD Putri SMD Ferandra VA The Evaluation Results of Proposed Antigen Rapid Diagnostic Tests for COVID-19: Some Possible Factors Might Influence. Infection (2023) 2023:1–7. 10.1007/s15010-022-01975-9 PMC9806449 36592297

[61] Leixner G Voill-Glaninger A Krejci I Gaugeler-Kurzweil J Kusstatscher T Krugluger W Performance Study of the Anterior Nasal AMP SARS-CoV-2 Rapid Antigen Test in Comparison with Nasopharyngeal rRT-PCR. Access Microbiol (2022) 4(6):acmi000361. 10.1099/acmi.0.000361 36004360 PMC9394671

[62] Kawser Z Hossain M Suliman S Lockman S Gitaka J Bandawe G An Assessment of a Rapid SARS-CoV-2 Antigen Test in Bangladesh. Am J Trop Med Hyg (2022) 107(4):845–9. 10.4269/ajtmh.22-0068 35970285 PMC9651516

[63] Eikelenboom-Boskamp A den Ouden M de Groot T Stobernack T Wertheim H Voss A. Evaluation of the Abbott Panbio™ COVID-19 Antigen Detection Rapid Diagnostic Test Among Healthcare Workers in Elderly Care. PLoS One (2023) 18(2):e0276244. 10.1371/journal.pone.0276244 36827362 PMC9955641

[64] Irungu JK Munyua P Ochieng C Juma B Amoth P Kuria F Diagnostic Accuracy of the Panbio COVID-19 Antigen Rapid Test Device for SARS-CoV-2 Detection in Kenya, 2021: A Field Evaluation. PLoS One (2023) 18(1):e0277657. 10.1371/journal.pone.0277657 36696882 PMC9876661

[65] Narumi N Kondo T Sato Y Katayama Y Nirasawa S Saeki M Analysis of Diagnostic Performance and Factors Causing Nonspecific Reactions in SARS-CoV-2 Rapid Antigen Detection Tests. J Infect Chemother (2023) 29(2):157–62. 10.1016/j.jiac.2022.10.007 36288777 PMC9595385

[66] Ristić M Nikolić N Čabarkapa V Turkulov V Petrović V. Validation of the STANDARD Q COVID-19 Antigen Test in Vojvodina, Serbia. PLoS One (2021) 16(2):e0247606. 10.1371/journal.pone.0247606 33617597 PMC7899368

[67] So MK Chung HS Lee DH Lee M. How Significant are Xpert Xpress SARS-CoV-2 Test Findings When Only an N2 Gene is Detected? Diagnostics (Basel) (2022) 12(9):2133. 10.3390/diagnostics12092133 36140534 PMC9498257

[68] Shen HC Hu YC Chen YF Tung TH. Prevalence and Associated Metabolic Factors of Gallstone Disease in the Elderly Agricultural and Fishing Population of Taiwan. Gastroenterol Res Pract (2014) 2014:876918. 10.1155/2014/876918 24707283 PMC3953423

[69] Tom MR Mina MJ. To Interpret the SARS-CoV-2 Test, Consider the Cycle Threshold Value. Clin Infect Dis (2020) 71:2252–4. 10.1093/cid/ciaa619 32435816 PMC7314112

[70] Romero-Gómez MP Gómez-Sebastian S Cendejas-Bueno E Montero-Vega MD Mingorance J García-Rodríguez J SARS-CoV-2 Working Group. Ct Value is not Enough to Discriminate Patients Harbouring Infective Virus. J Infect (2021) 82:e35–e37. 10.1016/j.jinf.2020.11.025 PMC7688433 33248218

[71] Fujita-Rohwerder N Beckmann L Zens Y Verma A. Diagnostic Accuracy of Rapid Point-Of-Care Tests for Diagnosis of Current SARS-CoV-2 Infections in Children: A Systematic Review and Meta-Analysis. BMJ Evid Based Med (2022) 27:274–87. 10.1136/bmjebm-2021-111828 PMC8783973 35042748

[72] Huff HV Singh A. Asymptomatic Transmission During the Coronavirus Disease 2019 Pandemic and Implications for Public Health Strategies. Clin Infect Dis (2020) 71:2752–6. 10.1093/cid/ciaa654 32463076 PMC7314132

[73] Ahmed N Kalil MNA Yusof W Bakar MAA Sjahid AS Hassan R A Performance Assessment Study of Different Clinical Samples for Rapid COVID-19 Antigen Diagnosis Tests. Diagnostics (Basel) (2022) 12(4):847. 10.3390/diagnostics12040847 35453895 PMC9030639

[74] Mayor S. Covid-19: Warning Over Transmission Risk as Self-Isolation is cut to Five Days in England. BMJ (2022) 376:o111. 10.1136/bmj.o111 35031534

[75] Mina JM Parker R Larremore BD. Rethinking Covid-19 Test Sensitivity - a Strategy for Containment. N Engl J Med (2020) 383(22):e120. 10.1056/NEJMp2025631 32997903

[76] Larremore DB Wilder B Lester E Shehata S Burke JM Hay JA Test Sensitivity is Secondary to Frequency and Turnaround Time for COVID-19 Surveillance. Sci Adv (2020) 7(1):2020.06.22.20136309. 10.1101/2020.06.22.20136309 PMC7775777 33219112

[77] Tolentino VR Derevlany L DeLaMothe C Vick S Chalyavski L. The Effects of the COVID-19 Pandemic on Risk Management Practice: A Report From the Epicenter of the Epicenter in New York City. J Healthc Risk Manag (2021) 40(4):46–57. 10.1002/jhrm.21461 33496013 PMC8013960

[78] Wu X Zhou H Wu X. Strategies for Qualified Triage Stations and Fever Clinics During the Outbreak of COVID-2019 in the County Hospitals of Western Chongqing. J Hosp Infect (2020) 105(2):128–9. 10.1016/j.jhin.2020.03.021 32205161 PMC7118631

[79] Yu X Yang R. COVID-19 Transmission Through Asymptomatic Carriers is a challenge to Containment. Influenza Other Respir Viruses (2020) 14(4):474–5. 10.1111/irv.12743 32246886 PMC7228388

[80] Olalekan A Iwalokun B Akinloye OM Popoola O Samuel TA Akinloye O. COVID-19 Rapid Diagnostic Test Could Contain Transmission in Low- and Middle-Income Countries. Afr J Lab Med (2020) 9(1):1255. 10.4102/ajlm.v9i1.1255 33102170 PMC7567180

[81] Arshadi M Fardsanei F Deihim B Farshadzadeh Z Nikkhahi F Khalili F Diagnostic Accuracy of Rapid Antigen Tests for COVID-19 Detection: A Systematic Review with Meta-Analysis. Front Med (Lausanne) (2022) 9:870738. 10.3389/fmed.2022.870738 35463027 PMC9021531

[82] Khandker SS Nik Hashim NHH Deris ZZ Shueb RH Islam MA. Diagnostic Accuracy of Rapid Antigen Test Kits for Detecting SARS-CoV-2: A Systematic Review and Meta-Analysis of 17,171 Suspected COVID-19 Patients. J Clin Med (2021) 10(16):3493. 10.3390/jcm10163493 34441789 PMC8397079

[83] Sedgwick P. Meta-analyses: What is Heterogeneity? BMJ (2015) 350:h1435. 10.1136/bmj.h1435 25778910

